# The role of early detection and treatment in malaria elimination

**DOI:** 10.1186/s12936-016-1399-y

**Published:** 2016-07-15

**Authors:** Jordi Landier, Daniel M. Parker, Aung Myint Thu, Verena I. Carrara, Khin Maung Lwin, Craig A. Bonnington, Sasithon Pukrittayakamee, Gilles Delmas, François H. Nosten

**Affiliations:** Shoklo Malaria Research Unit, Mahidol-Oxford Tropical Medicine Research Unit, Faculty of Tropical Medicine, Mahidol University, Mae Sot, Thailand; Faculty of Tropical Medicine, Mahidol University, Bangkok, Thailand; Centre for Tropical Medicine and Global Health, Nuffield Department of Medicine, University of Oxford, Oxford, UK

## Abstract

Falciparum malaria persists in hard-to-reach areas or demographic groups that are missed by conventional healthcare systems but could be reached by trained community members in a malaria post (MP). The main focus of a MP is to provide uninterrupted and rapid access to rapid diagnostic tests (RDTs) and artemisinin-based combination therapy (ACT) too all inhabitants of a village. RDTs allow trained community members to perform malaria diagnosis accurately and prescribe appropriate treatment, reducing as much as possible any delay between the onset of fever and treatment. Early treatment with ACT and with a low-dose of primaquine prevents further transmission from human to mosquito. A functioning MP represents an essential component of any malaria elimination strategy. Implementing large-scale, high-coverage, community-based early diagnosis and treatment through MPs requires few technological innovations but relies on a very well structured organization able to train, supervise and supply MPs, to monitor activity and to perform strict malaria surveillance.

**Electronic supplementary material:**

The online version of this article (doi:10.1186/s12936-016-1399-y) contains supplementary material, which is available to authorized users.

## Background

Despite recent significant progresses towards elimination in several countries in Asia, Africa and South America, malaria remains a major health issue in many tropical regions where it thrives in countries with a weak healthcare system [1]. In the last 20 years significant increases in investment have resulted in the development of new tools to combat this parasitic disease, the most common of mankind. Some of the newest tools require expensive and complex technologies that are not available to national malaria programmes, or are still under trial (vaccines, insecticides, genetically modified mosquitoes) [2]. Others are already available, such as long-lasting insecticidal bed nets (LLINs), rapid diagnostic tests (RDTs) and artemisinin-based combination treatment (ACT). Their large-scale deployment probably explains most of the decrease in malaria-related morbidity and mortality in Southeast Asia and Africa as well as renewed interest in malaria elimination [3–5]. However, in order to control malaria (reduction of morbidity and mortality) and eliminate it (interruption of the transmission cycle), it is essential to identify and treat infected individuals early in the course of the illness. To achieve this goal, everyone living in malaria-endemic areas must have easy access to reliable diagnostics and effective treatment. This is one of the most difficult tasks encountered by national malaria programmes.

The aim of the present article is to discuss in detail the concept of early diagnosis and treatment (EDT) of falciparum malaria cases. First, the rationale for community-based EDT and the concept of the malaria post (MP) are presented. Second, the novelties in tools and approaches for the successful deployment of MPs are detailed. Third, evidence of the impact of widespread deployment of community-based EDT is described, followed by the requirements for regional integration of an MP network as part of a falciparum malaria elimination strategy. Finally, an outline of the challenges to be met to ensure the sustainability of EDT by MPs is provided.

### Rationale for community-based EDT

EDT provides a means of decreasing transmission of *Plasmodium falciparum* from symptomatic individuals. In *P. falciparum* infections symptoms usually occur when the density of asexual parasites reaches a pyrogenic threshold, approximately 12–14 days after an infective bite [1]. Mature gametocytes are the sexual stages that are infective for mosquitoes. They emerge seven to 15 days after the onset of symptoms [6]. Treating *P. falciparum*-infected patients within 24–48 h after fever onset is likely to prevent further transmission of the parasite. Conversely, patients who delay treatment are more likely to remain infectious even after being treated because gametocytes may persist for several weeks after clearance of asexual parasites [7, 8]. Patients who experience a recurrence of their infection because they received an ineffective or incomplete treatment have higher gametocyte carriage and contribute disproportionately to transmission [9].

### The malaria post

Most healthcare delivery systems have a concentric structure centred on a referral hospital. At the periphery are dispensaries or health posts where people from surrounding villages go when they are ill. But individuals living in endemic regions face many barriers to accessing malaria diagnosis and treatment because of geographic distances or travel difficulties, as well as socio-cultural, economic, political, and legal factors [10–14]. Most of these problems can best be addressed through diagnosis and treatment based within the community [10, 15–17].

**Fig. 1:**
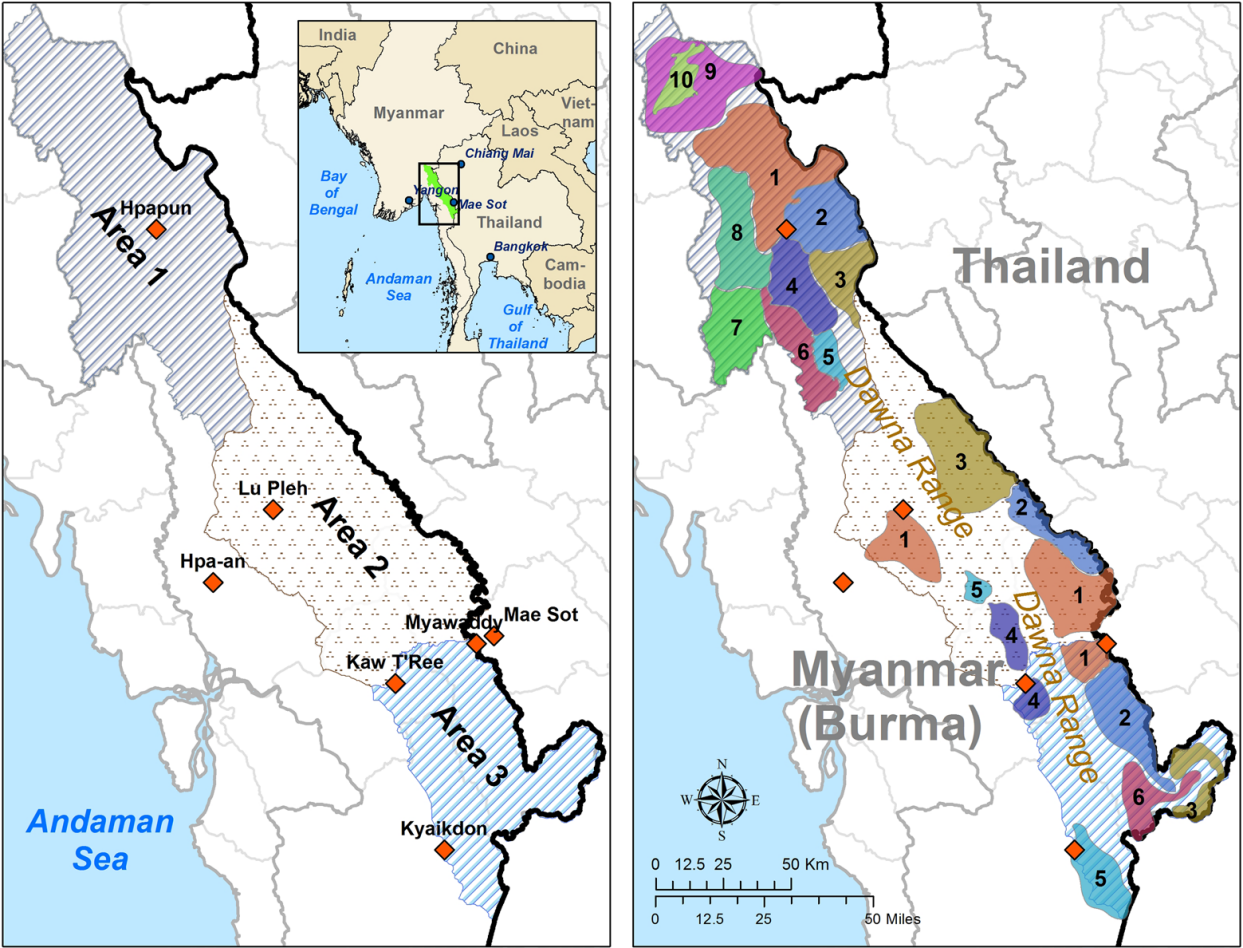
Map of the region of Eastern Myanmar targeted for large scale deployment of community-based EDT by the Malaria Elimination Task Force (METF). Drug and multidrug-resistant falciparum malaria is a major threat in the Thailand-Myanmar border region. In the past drug-resistant malaria has emerged in this area, and other parts of Southeast Asia, and subsequently spread globally. Elimination of falciparum malaria in the region is the only solution to avoid a repeat of history with artemisinin resistance. With this goal in mind, the METF was set up in 2014 and targets over 1200 villages in Eastern Kayin State, Myanmar [18]. The high-level divisions (area, zone) are represented. Each zone corresponds to a territory under the responsibility of one health community-based organization

Community-based EDT for malaria is implemented through a simple structure: the malaria post (MP). An MP is defined by three main criteria: (1) trained personnel; (2) quality RDT; and, (3) effective anti-malarial drugs. The MP is a simple structure that provides continuous access to reliable diagnosis and effective treatment for any clinical malaria cases from the community within 24–48 h of fever onset. The minimalistic nature of the MP makes it feasible to set up and maintain in difficult settings where malaria persists. An effective MP presents several important particularities: it offers free services and continuous availability of staff and supplies; it ensures diagnosis and treatment of clinical malaria; and it is part of a dense network designed to have an impact on malaria at the regional level. These attributes allow large-scale implementation of efficient malaria EDT by MP in almost any setting. A network of MP is the necessary backbone on which to build a *P. falciparum* malaria elimination programme (Fig. 1). MP should be located within an easy walking distance (e.g., 15 min) from any home, keeping in mind that a short geographical distance is a necessary factor for access, but not sufficient if social, behavioural and other barriers are overlooked.

### Costs and cost-effectiveness of MP for malaria elimination

**Fig. 2:**
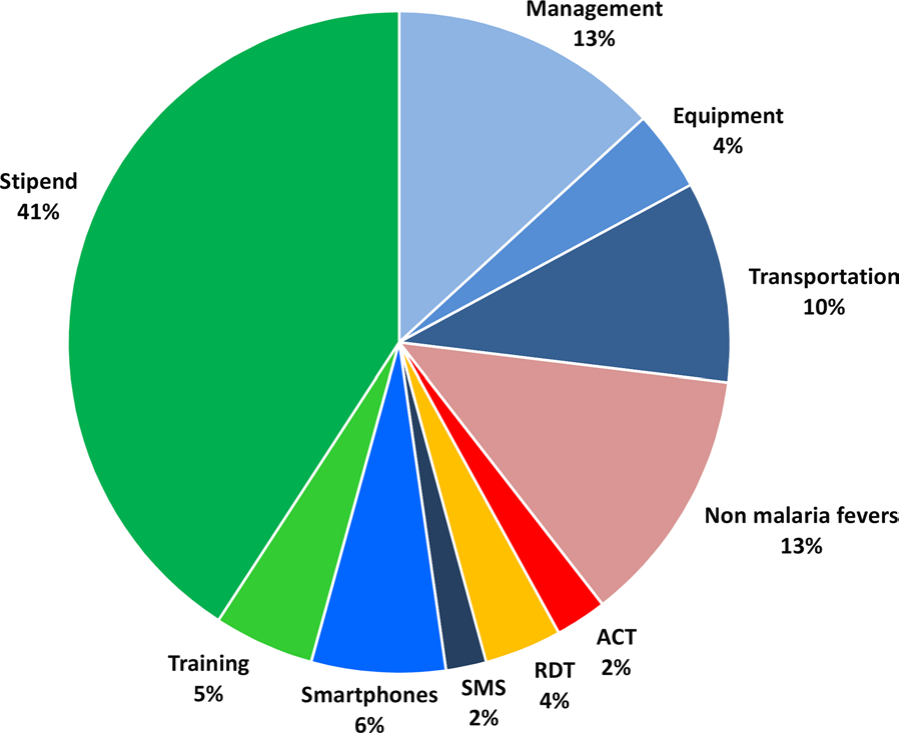
Distribution of monthly costs in an METF-supported MP. Costs of METF MP are around US$160, similar to other programs in Myanmar [20]. The costs of training, transportation (supervision, supply, patient referrals), data transmission (smartphone and SMS) and management of the MP network (36 % of total), emphasize the necessary investment on constant availability of supplies, regular monitoring and weekly malaria surveillance in MP. Stock-outs, lack of refresher trainings and/or monitoring visits have been reported as major risks for losing the trust of the communities and demotivation of workers in other programs of malaria EDT [40, 41], and are a major threat to elimination programs (see also “Challenges” section)

Apart from ACT and RDTs, an MP requires very little equipment: pregnancy tests and antimalarial treatment for pregnant women, paracetamol for non-malaria fever, a watch or clock to ensure timely reading of RDT and a scale for ACT dosing (see Additional file 1 for a complete list of supplies). MPWs should be provided with a reference handbook, including drug-dosing tables and decision algorithms. Used RDTs should be collected monthly for quality control and adequate disposal. In the large-scale deployment of EDT in Eastern Myanmar realized by the METF (Fig. 1), the monthly cost of an MP is around US$160, including the management of the MP network (Fig. 2) [18]. Community-based treatment, LLIN deployment and combined strategies have been assessed in a modelling work in the context of Southeastern Myanmar. In a malaria control perspective, the combined strategy was the most cost-effective, especially in communities where access to healthcare was difficult, even though transportation costs increased [19]. In METF, costs of MP are compatible with estimated values of standard community health workers in Myanmar, although the distribution of these costs differs significantly (Fig. 2) [20].

### New tools and approaches driving MP efficacy

#### RDTs

In the 1950s, pioneering programmes in Latin America and Southeast Asia relied on village-based volunteers to collect blood smears of suspected cases. Since microscopy was the only diagnosis confirmation available, volunteers had to provide presumptive treatment or to refer to a central or mobile microscopy-equipped facility for diagnosis confirmation, resulting in delayed treatment [21]. Populations living in remote areas were frequently neglected. Today RDTs overcome many bottlenecks encountered by microscopy: it is faster to train unskilled personnel to use RDTs than to train a good microscopy technician; there is no ‘fatigue’ or loss of blood smear reading skills because of a rarity of positive cases in a context of decreasing incidence; and, RDTs are easy to supply [22, 23]. Recent systematic reviews have concluded that RDT interpretation and performance are satisfactory when handled by trained community members and can result in the appropriate prescription of ACT for confirmed *P. falciparum* cases [24–26]. Recent improvements in RDT sensitivity and specificity mean that they perform as well as microscopy for symptomatic falciparum malaria infections in many settings [27]. RDTs can reliably discriminate between *P. falciparum* and *P. vivax* infections [22, 28, 29]. The good performances achieved by RDTs should be taken as strong encouragement to further develop this tool. For example, it is crucial to work on the detection of *P. falciparum* parasites lacking PfHRP2. These parasites are of concern in South America and should be monitored elsewhere to ensure the sustained efficacy of the RDT in use [30]. Additionally, it is important to improve the specifications of RDTs so that results can remain readable over a longer time period to facilitate monitoring of activities and quality control.

#### ACT and low-dose primaquine

ACT rapidly kills asexual stage *P. falciparum* parasites, preventing gametocytogenesis [31, 32]. Artemisinins are also active against stages I–IV gametocytes but not against the mature infectious gametocytes (stage V) [9, 33–35]. These forms develop in patients who present late for treatment, making such patients important sources of transmission [31]. Primaquine is the only currently available anti-malarial active against mature *P. falciparum* gametocytes [36]. The single, low dose recommended by WHO is unlikely to cause severe haemolytic anaemia even in individuals with G6PD deficiency [37]. When administered early in the course of the disease in combination with an ACT it shows a dramatic effect on transmissibility of *P. falciparum* [36, 38].

In many regions, the efficacy of malaria treatment is threatened by the use of counterfeit or sub-standard drugs [39]. Widely available quality drugs limit the number of patients exposed to sub-therapeutic doses of anti-malarials, contributing to the fight against the emergence and the selection of drug-resistant parasites.

#### Trained and supported MP workers

Malaria post workers (MPW) should be members of the community that they will serve, and should be selected by their peers. They should have at least some basic education and should have previously shown some interest in health-related work. MPWs must have an occupation that does not require them to leave their village for more than a day at a time. Their training should include an introduction to malaria, the use of RDT and ACT, when to refer patients, and the recording and reporting of their activities (Table 1). In order to maintain an adequate standard of care, close supervision and regular quality control of RDT results are necessary, especially during the initial weeks and months after an MP opening [40, 41]. This allows for retraining or replacement of MPWs if necessary. MPWs should receive fair compensation for their activities and constant availability [26]. Community-based programmes often rely on unpaid volunteers or provide activity-based compensation (e.g., per consultation or per RDT) but these types of incentives are not adapted to a context of highly seasonal illness or expected decreases in malaria incidence throughout an effective elimination programme. While some individuals may find it extremely rewarding to provide health services to their community on a voluntary basis [25, 40], remuneration of MPWs can promote objective-driven motivation and drastically reduce attrition and corruption.

**Table 1:** Malaria post worker (MPW) training and tasks

Training
Recognize signs of malaria
Conduct and interpret rapid test results
Observe universal precautions
Record, report and transfer results (weekly)
Knowledge on where to refer severe cases (local clinic)

Tasks
Stay in the village and be able to treat malaria within 24 h of fever onset. Make sure village population is aware of the existence of the MP
Administer drugs to patients according to protocol (including adherence monitoring if not directly observing treatment)
Monitor patient’s condition and recognize side effects
Refer severe cases and possible treatment failures to nearest health centre
Weekly check stocks and transmit appropriate orders to supervisor
Keep working environment clean and safe
Detect and report all malaria cases to supervisor
Engage community on malaria-related public health issues (e.g., use of LLIN or other vector control)

In some settings, private drug retailers might already exist in a community. In a context of malaria elimination, provision of free EDT to rare malaria cases will not represent a significant loss of income to such drug retailers. If they provide a valuable health service, it is desirable that this service should be monitored and quality assured. As any other member of the community meeting the requirements, they can be appointed as MPWs if they agree to be trained and to follow MP principles.

#### Community engagement

Taking into account local context is crucial to setting up a quality service that becomes a long-term part of community life [42]. This observation is not new but increased awareness that village-based EDT programmes will fail unless there is acceptance and support from the population has led to the promotion of active engagement strategies [43]. The community engagement (CE) team must have a deep understanding of the local population and develop and maintain trust with the community. They should meet with the people, their leaders and representatives in order to explain the objectives, technicalities and benefits of the MP. Unlike past attempts at community-based malaria interventions, the MP can provide care to uncomplicated malaria cases without delay or referral [21]. Entrusting this responsibility to the community represents a significant step of empowerment especially for vulnerable or marginalized populations.

### Large-scale deployment of community-based EDT for falciparum elimination

#### Evidence of the impact of the deployment of community-based EDT with ACT

Reports from a wide range of spatial and demographic scales in various geographic settings describe downward trends in clinical falciparum malaria incidence after the deployment of EDT using ACT and RDTs over the last 15 years [3, 4, 44]. Monitoring in settings where EDT has been consistently available over long periods of time suggests that EDT in combination with vector control measures can achieve near-elimination of falciparum malaria. On the Thai-Myanmar border, 15 years of EDT with ACT in clinics serving migrant and displaced populations drastically reduced the incidence of falciparum malaria [45, 46]. Malaria elimination efforts in Thailand were largely effective for much of the central plains region of the Kingdom but deteriorated with distance [47]. Tak Province is an example of a remote malarious region along the Thailand-Myanmar border. In 2001–2002 a community-based EDT strategy (The Tak Malaria Initiative) was deployed in all five sub-districts of the province [48]. In addition to the 92 existing provincial health facilities, 100 community-based malaria treatment posts were set up in villages and areas where access to health services was low. Over the following 2 years clinical *P. falciparum* incidence was reduced by 54 %, malaria-related deaths by 52 % and *P. falciparum* prevalence was significantly lower in intervention villages in comparison to controls [48]. Malaria vectors were still present but the entomological inoculation rate (EIR) was very low [48]. In Asia, similar declines in morbidity and mortality were reported from Cambodia after country-wide implementation [49]. Reports of ACT deployment on the African continent are equally encouraging: in the quasi-experimental setting of Dielmo village in Senegal, deployment of ACT at a local clinic in 2006 and mass distribution of LLINs in 2008 impacted both *P. falciparum* incidence and prevalence so drastically that in 2012 malaria was nearly eliminated [50]. Deployment of community-based EDT was also followed by decreases in *P. falciparum*-related morbidity, mortality and re-infection in clinical trials [24–26]. After regional implementation, similar decreases in incidence of morbidity and mortality were observed in Kwazulu-Natal [44] and a decreased prevalence of *P. falciparum* infection was reported from Ethiopia [5].

#### Requirements for regional integration in an elimination strategy

Even if a significant impact can be measured at village scale, the largest and longest term impacts towards elimination can only be achieved if the MP programme is deployed in all villages of a region (i.e., a geographical unit defined by a population, political boundaries and accessibility criteria). Coordination at regional (province, district, state) and central administrative levels of a country offers a wide geographical spread, the ability to take local specificities into account (different seasonality, for example) with relevant administrative divisions to interface with the health system in place. It is, however, advisable to always adapt divisions of a programme to local constraints, especially in peripheries and on borders where the population is usually less well covered.

##### Exhaustive mapping of communities in target region

Detailed data on the distribution of human settlements across malarious landscapes are frequently lacking making it difficult or impossible to know the true malaria burden and or the healthcare needs. Systematic geographic and demographic surveys are therefore a prerequisite to acquire accurate baseline data. Prior to the deployment of a network of MP, a detailed map of all communities in the target region must be drawn with geographic coordinates and estimated numbers of households for all settlements, the location of existing health facilities and access to communication networks (Fig. 1). This step is crucial to ensure that MPWs are deployed in all human settlements and not according to perceived access or other biased measures. It also allows for documentation of higher-level health structures on which the MP support system can rely and to build a geographic information system to follow the implementation of the programme and its efficiency.

##### Malaria surveillance and MP activity monitoring through real-time data reporting

Reporting of cases and inventory is key for surveillance and logistics. Information can be collected quickly on smartphones using dedicated entry forms, and transmitted effortlessly through internet or SMS in regions where phone network is available [51, 52]. In regions lacking a phone network, paper data sheets can be effectively collected and transported to central collection and data entry points. Weekly analysis of malaria incidence reports allows identification of communities where falciparum malaria is not following the expected decreasing trends. Spatio-temporal analysis of weekly incidence reports allows regional-scale assessment of villages or clusters of villages where persisting or higher-than-expected *P. falciparum* incidence is detected and to adjust the magnitude of targeted interventions in response [53, 54]. Analysis of weekly MP reports also provides alerts on possible MP malfunction or interruption of service (missing reports, lack of activity, supply shortage, etc.), which is used to target supervision visits.

##### Integration within the regional health system network

In many nations the health care system does not reach all communities. The MP network is specifically designed to address this critical gap, while avoiding redundancies. If there are available healthcare units (e.g., health clinics), these can be used as focal points for MP located in surrounding villages. They will be the referral centre for patients that cannot be treated at MP. They can also act as the last logistic node: dispatch MP supplies, collect and transmit weekly data reports. The medical staff can supervise MPW activities. At higher levels, health districts and sub-districts can handle the supervision of the network (MPW training, MPW monitoring and supervision) and its management (logistic, financial, administrative). Likewise, data collection and surveillance tasks can be integrated within an existing health management information system (HMIS) if it is able to sustain the frequency of reporting and timeliness required for malaria elimination surveillance. Alternatively, a specific system can be set up. As it is relying on high-resolution spatial and temporal data, it can easily be interfaced with existing reporting frameworks and can also become the backbone of a future HMIS.

### Challenges

#### Maintaining the MP and the network

The main challenges are to continuously maintain MP supplies, to ensure regular collection of accurate and complete data records and to monitor the activities of MPWs and the quality of services, as was recently described in studies of village health workers performing malaria EDT in Myanmar [40, 41]. Real-time, spatially explicit monitoring of activity and surveillance, as described in the previous section, will only be relevant if a strong organization with deep local roots and a dense network is acting in the field to conduct routine activities and follow-up on alerts. This organization can merge with and benefit from existing health structures where possible. Community engagement at regional level will involve coordination with community-based organizations, governmental and traditional authorities.

Vertical malaria-centred treatment is necessary to properly address persistent falciparum malaria in places where it has otherwise been difficult or impossible to eliminate. Over 90 % of malaria resurgence events were at least partly associated with failure to maintain malaria control programme interventions [55]. MPWs may lose focus, motivation or become complacent when falciparum malaria cases become increasingly rare [40]. MP will remain at the centre of the elimination strategy in regions where *P. vivax* is also present. In other areas it may be necessary to switch to integrated community case management (ICCM) of additional diseases in order to retain the focus on EDT for all potential malaria patients [40]. ICCM can increase public health relevance and cost effectiveness of the MP in a malaria elimination setting, where costs per case treated by an MP are expected to rise as malaria cases decrease [56, 57]. ICCM represents an additional step in community-based health provision and new activities can benefit from training, supervision, reporting, and logistic backbone already implemented for malaria elimination.

#### *P. falciparum* drug resistance

Malaria has decreased several times before in history and gains were lost when drug-resistant *P. falciparum* arose [45, 50, 58]. The development of chloroquine resistance in falciparum malaria and its subsequent spread globally inflicted a catastrophic blow to elimination efforts. Collection of *P. falciparum*-positive RDTs or additional collection of dried blood spots from all MP or from a selection of sentinel sites, allows the monitoring of markers of resistance to artemisinin (K13 polymorphism) as well as partner drugs (e.g., PfMDR1 for mefloquine/lumefantrine) [59–61]. Treatment and containment strategies can then be developed and implemented based on the drug resistance surveillance network.

#### Vivax malaria

If complete malaria elimination is the goal, vivax malaria must be eliminated in regions where it is prevalent. A different approach from that used for *P. falciparum* elimination is required because of differences in the life cycle of the two parasites. A typical MP is only equipped to treat the asexual blood stages of *P. vivax*. *Plasmodium vivax* gametocytes emerge much earlier in the course of an infection, sometimes prior to clinical presentation. EDT is therefore not expected to be as efficient at breaking the transmission cycle of *P. vivax* compared with *P. falciparum* [8, 62]. *Plasmodium vivax* is characterized by liver-stage hypnozoites which cause relapsing blood-stream infections without exposure to new infective bites. These hypnozoites are not killed by chloroquine and relapses are therefore not prevented by early treatment at the MP [63]. The only available treatment for the radical cure for *P. vivax* is a 14-day course of primaquine but it is rarely used because of poor adherence and potential toxicity in case of G6PD deficiency [64, 65]. Point-of-care G6PD tests and better treatments of the hypnozoites are needed to eliminate *P. vivax*.

## Conclusions

In the context of rising artemisinin- and multidrug-resistance, significant gains can still be achieved by making EDT available to everyone. The MP is a simple structure relying on three equally crucial components to provide EDT: RDTs, ACT+ primaquine, and trained and paid MPWs originating from the community. Setting up, monitoring, supplying, and collecting activity data from MP in the hardest-to-reach malaria endemic areas represents a logistic and organizational challenge but will yield a major impact. A coordinated MP network can be expected to trigger a regional rapid decrease in falciparum malaria clinical case incidence and prevalence, a highly desirable outcome in a malaria elimination strategy. In addition to this generalized MP coverage, elimination could be quickened by a limited number of targeted interventions, such as mass-drug administration and adapted vector control in high-risk communities.

## Additional file

10.1186/s12936-016-1399-y Supply list for opening a new malaria post, following the example of a 200-inhabitant community within METF programme. METF is operating in Eastern Myanmar, which is characterized by seasonal malaria with both *P. falciparum* and *P. vivax* parasites. In METF, MP use of consumables is assessed every week so that it can be restocked appropriately. MP data are checked and collected weekly by a supervisor, who then transmits using an SMS-based data reporting system where a phone-network service is available [18]. Quantity and type of supplies should be adapted to each setting according to malaria incidence rate and supply chain constraints, and following national malaria control programme guidelines and international recommendations. For example, RDT can be any RDT in the recommended procurement list of the WHO/GMP.

## Abbreviations

ACT: artemisinin-based combination treatment
CE: community engagement
EDT: early diagnosis and treatment
EIR: entomological inoculation rate
G6PD: glucose-6-phosphate dehydrogenase
HMIS: health management information system
ICCM: integrated community case management
LLIN: long-lasting insecticidal net
METF: Malaria Elimination Task Force
MP: malaria post
MPW: malaria post worker
RDT: rapid diagnostic test
SMS: short messaging system
