# Supplementary material for: The role of early detection and treatment in malaria elimination
Source: Malar J. 2016 Jul 15;15:363. doi: 10.1186/s12936-016-1399-y (PMC4946177; doi:10.1186/s12936-016-1399-y)
Supplement: Supplementary file 1 — 10.1186/s12936-016-1399-y Supply list for opening a new malaria post, following the example of a 200-inhabitant community within METF programme. METF is operating in Eastern Myanmar, which is characterized by seasonal malaria with both P. falciparum and P. vivax parasites. In METF, MP use of consumables is assessed every week so that it can be restocked appropriately. MP data are checked and collected weekly by a supervisor, who then transmits using an SMS-based data reporting system where a phone-network service is available [18]. Quantity and type of supplies should be adapted to each setting according to malaria incidence rate and supply chain constraints, and following national malaria control programme guidelines and international recommendations. For example, RDT can be any RDT in the recommended procurement list of the WHO/GMP. [file 12936_2016_1399_MOESM1_ESM.docx]

| **Items** | **Example for METF Malaria Post in a 200-inhabitant community** | |
| --- | --- | --- |
| **Forms** | **Quantity** | **Specification** |
| Daily forms (patients log) | 50 | Sheets |
| Weekly aggregate data forms | 12 | Sheets |
| Malaria case logbook | 1 | 30-page printed notebook |
| **Malaria Drugs** |  |  |
| *P. vivax* treatment | 50* | 300 Tablets Chloroquine 250 mg |
| *P. falciparum* treatment | 30* | 720 Tablets Coartem® (Artemether 20 mg+ Lumefantrine 120 mg) |
| *P. falciparum* treatment for 1^st^ trimester pregnant women | 5* | 42 tablets: Clindamycin 300 mg  42 tablets: Quinine 300 mg |
| Treatment of *P. falciparum* gametocytes (preventing transmission) | 50* | 100 tablets Primaquine 7.5mg |
| **Other Drugs** |  |  |
| Paracetamol 500 mg | 1000 | Tablets |
| Ferrous sulphate + Folic Acid | 1000+1000 | Tablets |
| **Diagnostic Tests** |  |  |
| RDT *P. falciparum*/*P. vivax* | 100 | Tests |
| Pregnancy Test kits | 10 | Tests |
| **Other medical supplies** |  |  |
| Thermometer | 2 | Pcs. |
| Tablet cutter | 1 | Pcs. |
| Gloves | 1 | box |
| Soap | 2 | Pcs |
| Scissor | 1 | Pcs |
| Weighing scales (in kilos) | 1 | Pcs. |
| Alcohol 240mL | 1 | Bottle |
| Cotton roll + Gauze roll | 1 + 1 | Pcs |
| **Stationary & Others** |  |  |
| Pens (black+red) | 2 | Pcs. |
| Permanent markers for RDT (black/red) | 3/1 | Pcs. |
| pencils | 1 | Pcs. |
| rubber/eraser | 1 | Pcs. |
| ruler | 1 | Pcs. |
| Wall clock | 1 | Pcs. |
| Calculator | 1 | Pcs. |
| Plastic folders | 2 | Pcs. |
| plastic bags (RDT storage) | 1 | Pack |
| Rubbish bag (black) | 12 | Pcs. |
| Used needle bin/Sharp bin (Red) | 1 | Pcs. |
| Notebook | 1 | Pcs. |
| Carbon paper | 10 | Sheets |
| Stapler+ staples | 1 | Pcs. |
| File (Folder binding) | 1 | Pcs. |
| Plastic Box | 1 | Pcs. |

* Indicative number of treatments as drugs are administered according to body weight following dosing charts.
